# Benzyl isothiocyanate (BITC) triggers mitochondria-mediated apoptotic machinery in human cisplatin-resistant oral cancer CAR cells

**DOI:** 10.1051/bmdcn/2018080315

**Published:** 2018-08-24

**Authors:** Chiu-Fang Lee, Ni-Na Chiang, Yao-Hua Lu, Yu-Syuan Huang, Jai-Sing Yang, Shih-Chang Tsai, Chi-Cheng Lu, Fu-An Chen

**Affiliations:** 1 Department of Pharmacy, Kaohsiung Veterans General Hospital Pingtung Branch Pingtung 912 Taiwan; 2 Department of Pharmacy and Master Program, Tajen University Pingtung 907 Taiwan; 3 Department of Medical Research, China Medical University Hospital, China Medical University Taichung 404 Taiwan; 4 Department of Biological Science and Technology, China Medical University Taichung 404 Taiwan; 5 Department of Pharmacy, Buddhist Tzu Chi General Hospital Hualien 970 Taiwan; 6 Department of Sport Performance, National Taiwan University of Sport Taichung 404 Taiwan

**Keywords:** Benzyl isothiocyanate (BITC), Human cisplatin-resistant oral cancer CAR cells, Apoptosis, Mitochondria

## Abstract

Benzyl isothiocyanate (BITC), a component of dietary food, possesses a powerful anticancer activity. Previous studies have shown that BITC produces a large number of intracellular reactive oxygen species (ROS) and increases intracellular Ca^2+^ release from endoplasmic reticulum (ER), leading to the activation of the apoptotic mechanism in tumor cells. However, there is not much known regarding the inhibitory effect of BITC on cisplatin-resistant oral cancer cells. The purpose of this study was to examine the anticancer effect and molecular mechanism of BITC on human cisplatin-resistant oral cancer CAR cells. Our results demonstrated that BITC significantly reduced cell viability of CAR cells in a concentration- and time-dependent manner. BITC was found to cause apoptotic cell shrinkage and DNA fragmentation by morphologic observation and TUNEL/DAPI staining. Pretreatment of cells with a specific inhibitor of pan-caspase significantly reduced cell death caused by BITC. Colorimetric assay analyses also showed that the activities of caspase-3 and caspase-9 were elevated in BITC-treated CAR cells. An increase in ROS production and loss of mitochondria membrane potential (ΔΨm) occurred due to BITC exposure and was observed *via* flow cytometric analysis. Western blotting analyses demonstrated that the protein levels of Bax, Bad, cytochrome *c,* and cleaved caspase-3 were up-regulated, while those of Bcl-2, Bcl-xL and pro-caspase-9 were down-regulated in CAR cells after BITC challenge. In sum, the mitochondria-dependent pathway might contribute to BITC-induced apoptosis in human cisplatin-resistant oral cancer CAR cells.

## 1. Introduction

Cruciferous vegetables, including cauliflower, cabbage, and broccoli have been found to prevent the development of cancer [1–3]. Once cruciferous vegetables are cut, crushed, or chewed, a special odor metabolite-isothiocyanate (ITC) is produced [4–6]. ITCs are derived from glucosinolates and are effective chemo-preventive agents in tissues and organs, including the lungs, esophagus, chest, liver, small intestine, large intestine, pancreas, and bladder, for carcinogen-induced cancer [4–6]. In addition, ITCs have been found to induce apoptosis and autophagy in various types of cancer cells [7–9]. Common ITC derivatives [allyl isothiocyanate (AITC), benzyl isothiocyanate (BITC), phenethyl isothiocyanate (PEITC), and sulforaphane (SFN)] have been shown to have anticancer cell growth and apoptosis-inducing effects [10–24]. However, what is not fully understood is the mechanism of action of ITC on human cisplatin-resistant oral cancer cells.

Evading apoptosis is defined as a hallmark of cancer, and its anti-apoptotic ability has emerged as a criterion for mainstream drug development [25–28]. Apoptosis signaling can be divided into two distinct pathways: The intrinsic and the extrinsic cell death signaling pathways [28]. The intrinsic machinery is triggered by cellular stresses through either mitochondria or endoplasmic reticulum, resulting in the alteration of the Bcl-2 family molecules and caspases proteins [29]. The Bcl-2 family members consists of three groups: Pro-apoptotic proteins (such as Bax and Bak), anti-apoptotic proteins (such as Bcl-2 and Bcl-xL), and Bcl-2 regulators (also known as BH3-only proteins) [29, 30]. Upon cell stimulation, anti-apoptotic proteins play the important roles of maintaining mitochondrial integrity and preventing cytochrome *c* release, while pro-apoptotic proteins move to the mitochondria and cause mitochondrial membrane potential changes, leading to cytochrome *c* release [28–31]. Cytochrome *c* and apoptotic protease-activating factor-1 (Apaf-1) form a complex called apoptosome [28, 30]. Apoptosome cleaves pro-caspase-9 and then activates downstream caspase-3, which leads to apoptosis. In addition, anti-apoptotic proteins block apoptosis-inducing factor (AIF), and endonuclease G (Endo G) release from the mitochondria into the cytosol. The release of both AIF and Endo G also causes DNA fragmentation and induces cell apoptosis [6, 8, 31].

The extrinsic pathway initiates the binding of extrinsic signals to the death receptors (DRs) [28, 32]. For example, Fas, a member of the tumor necrosis factor receptors (TNFRs), binds to Fas ligand (FasL) and recruits downstream the Fas-associated death domain (FADD), and this forms a death-inducing signaling complex (DISC) and activates caspase-8 [9, 33]. Caspase-8 activation turns on the downstream effector caspase-3 and induces apoptosis. TNFRs include TNFR1, DR3, DR4 (tumor necrosis factor-related apoptosis-inducing ligand receptor 1, TRIAL R1), DR5 (TRIAL R2), and DR6. Previous studies have shown that caspase-8 activation cleaves Bid (a pro-apoptotic protein) and blocks Bcl-2, which results in cytochrome *c* release and triggers apoptosis [32, 34, 35]. Therefore, a potential approach to fighting cancer cells may be through the induction of apoptotic signaling [28, 32, 34]. In the present study, we investigated the oral anticancer effect and the possible molecular mechanism of BITC-induced apoptosis on human cisplatin-resistant oral cancer CAR cells.

## 2. Materials and methods

### 2.1. Chemicals, reagents, and antibodies

Benzyl isothiocyanate (BITC), cisplatin, dimethyl sulfoxide (DMSO), 3-(4,5-dimethylthiazol-2-yl)-2,5-diphenyltetrazolium bromide (MTT), and other chemicals of analytical grade were acquired from Sigma-Aldrich (St. Louis, MO, USA) unless otherwise specified. Dulbecco’s modified Eagles medium (DMEM), fetal bovine serum (FBS), L-glutamine, and penicillin/streptomycin were purchased from HyClone (Logan, UT, USA). ZVAD-fmk (a pan-caspase inhibitor) was purchased from Merck Millipore (Billerica, MA, USA). Caspase-3 and Caspase-9 Colorimetric Assay Kits were obtained from R&D Systems (Minneapolis, MN, USA). 2’,7’-Dichlorodihydrofluorescein diacetate (H_2_DCFDA) (an ROS indicator) and 3,3’-dihexyloxacarbocyanine iodide [DiOC_6_(3)] [a mitochondrial membrane potential (Δψm) detector] were purchased from Molecular Probes/Thermo Fisher Scientific (Waltham, MA, USA). The anti-Bax, anti-Bad, anti- Bcl-2, anti-Bcl-xL, anti-cytochrome *c,* anti-caspase-9, anti-caspase-3, and anti-β-actin, as well as anti-rabbit IgG or anti-mouse horseradish peroxidase (HRP)-linked antibodies were all bought from GeneTex (Hsinchu, Taiwan).

### 2.2. Cell culture

The cisplatin-resistant oral cancer CAR cells were established *via* gradient induction of increasing concentrations (10-80 μM ) of cisplatin up to 80 μM in parental human tongue squamous cell carcinoma cell line CAL 27 (American Type Culture Collection, ATCC, Manassas, VA, USA), as previously described [36-38]. CAR cells were cultured in DMEM with 10% FBS, 2 mM L-glutamine, and 1% antibiotics (100 Unit/ml penicillin and 100 μg/ ml streptomycin) at 37°C in a 5% CO_2_ humidified incubator.

### 2.3. Cell viability *via* MTT assay

CAR cells were seeded in 96-well plates at a density of 1 × 10^4^ cells per well in 100 μl and then exposed to 0, 2.5, 5, 10, and 20 μM of BITC for 24 or 48 h before pre-incubation with or without 10 μM Z-VAD-fmk (a pan-caspase inhibitor) for 1 h. After that, cells were incubated with 0.5 mg/ml MTT solution for additional 2 h. The medium was removed, and 100 μl DMSO was added to dissolve the blue formazan. The optical density was measured at the absorbance of 570 nm using a spectrophotometer, as previously described [39].

### 2.4. Dynamic cell confluence assay

CAR cells (1 × 10^4^ cells per well) were plated in a 96-well plate and then treated with 0, 5, 10, and 20 μM of BITC for 48 h. The cell confluence experiment was conducted over 48 h using an IncuCyte ZOOM System instrument (Essen BioScience, Ann Arbor, MI, USA). Data collection was performed every 2 h, and the morphological image was recorded and photographed every 12 h, as previously described [38, 40].

### 2.5. TUNEL/DAPI staining

CAR cells (1 × 10^5^ cells/ml) in 12-well plates were harvested following treatment with 0, 2.5, 5, and 10 μM of BITC for 48 h. Cells were fixed in 100% methanol at room temperature for 10 min, and then stained with 4’-6-diamidino-2-phenylindole (DAPI) solution (1 μg/ml). DNA breaks were detected with an *In Situ* Cell Death Detection Kit, Fluorescein (Roche Diagnostics GmbH; Sigma-Aldrich) according to the manufacturer's instructions. Apoptotic cells were observed and photographed under a fluorescent microscope, as previously described [39].

### 2.6. Cell morphology changes

CAR cells (1 × 10^4^ cells/100 μl) in 96-well plates were treated with or without 10 μM BITC for 48 h after pre-incubation with 10 μM Z-VAD-fmk (a pan-caspase inhibitor) for 1 h. After that, cells were visualized and photographed under a phase-contrast microscope as previously described [27].

### 2.7. Colorimetric assays analyses of caspase-3/-9 activities

CAR cells (5 × 10^6^ cells per 75T flask) were treated with 0, 2.5, 5, and 10 μM of BITC for 48 h. Cell lysates were harvested, the supernatants were incubated with the supplied reaction buffer with dithiothreitol and DEAD-pNA (for caspase-3) or LEHD-pNA (for caspase-9) as substrates at 37°C for 2 h as per the manufacturer’s protocols (Caspase-3 and Caspase-9 Colorimetric Assay Kits, R&D System Inc., Minneapolis, MN, USA).

### 2.8. Assays of ROS production and mitochondrial membrane potential (ΔΨm) by flow cytometry

CAR cells (2 × 10^5^ cells/ml) in 12-well plates were incubated with 0, 2.5, 5, and 10 μM of BITC for 48 h. The cells were harvested and probed with 500 μl of 10 μM H2DCF-DA (an ROS dye) and 50 nM DiOC_6_(3) (a cell-permeant ΔΨm probe), respectively, for 30 min at 37°C by flow cytometry, as previously described [41, 42].

### 2.9. Western blotting

CAR cells (5 × 10^6^ cells per 75T flask) were treated with 0, 2.5, 5, and 10 μM of BITC for 48 h. The cells were harvested and lysed in the Trident RIPA Lysis Buffer (GeneTex), and the protein concentration was detected using a Pierce BCA protein assay kit (Thermo Fisher Scientific). An equal amount of the protein sample (40 μg) was resolved by a 10-12% SDS-PAGE, and then transferred to an Immobilon-P Transfer Membrane (Merck Millipore), as previously described [34, 42]. The membrane was incubated overnight with the following antibodies: Bax, Bad, Bcl-2, Bcl-xL, cytochrome *c*, caspase-9, and caspase-3 after being blocked with 5% skim milk for 1 h. The appropriate HRP-conjugated secondary antibodies were thereafter applied and incubated for 1 h to check the targeted protein using an Immobilon Western Chemiluminescent HRP Substrate (Merck Millipore), as previously described [43]. Densitometry analysis was performed using NIH ImageJ 1.47 software, and all bands were normalized to β-actin.

### 2.10. Statistical analysis.

Data are presented as the mean ± standard deviation (SD), and all experiments were performed in triplicate. All statistical analysis was assessed through one-way ANOVA using SPSS 14.0 software (SPSS, Inc., Chicago, IL, USA), followed by Dunnett's test. Any *P* value < 0.05 was considered to be statistically significant.

## 3. Results

### 3.1. BITC reduces the viability of human cisplatin-resistant oral cancer CAR cells

**Fig. 1:**
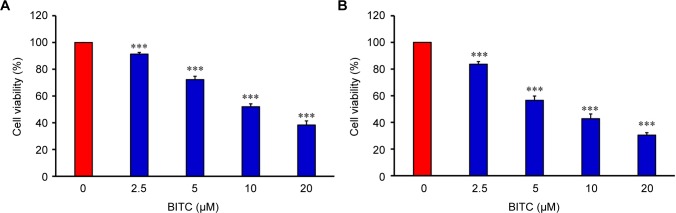
Effects of BITC on cell viability in CAR cells. Cells were placed in 96-well plates at a density of 1 × 10^4^ cells/well and were treated with 0, 2.5, 5, 10, and 20 μM of BITC for 24 (A) and 48 h (B). Cell viability was determined by an MTT assay. Each data point was shown as the means ± SD and independently repeated for three times. ***p < 0.001 compared with the untreated control group.

**Fig. 2:**
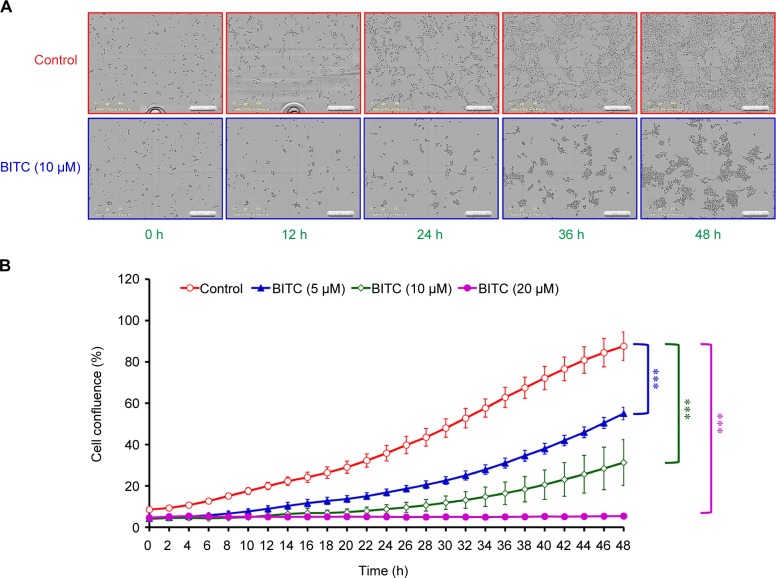
Effects of BITC on cell confluence of CAR cells. Cells were incubated with 0, 5, 10, or 20 μM of BITC for various durations. (A) Cell morphology and (B) cell confluence were determined by an IncuCyte ZOOM System instrument. Data are presented as the means ± SD (n = 3). ***p < 0.001 versus untreated control.

CAR cells were treated with different concentrations (0, 2.5, 5, 10, and 20 μM ) of BITC for 24 and 48 h, followed by an MTT assay. The results of this process indicated that BITC inhibited the cell growth of CAR cells in a concentration-dependent manner (Fig. 1A). Similarly, after 48 h exposure, BITC dramatically decreased CAR cell viability, and this effect was time- and concentration-dependent (Fig. 1B). Further analysis by cell confluence was performed using an IncuCyte ZOOM System instrument, where CAR cells after exposure to 0, 5, 10, and 20 μM of BITC were monitored up to 48 h. Our results demonstrated that BITC markedly suppressed the cell number after 12 h exposure, and the image was photographed at the 12-h mark in CAR cells (Fig. 2A). The inhibitory effect of CAR cell confluence was observed after BITC challenge, when compared to the control up to 48 h (Fig. 2B and Supplementary data). These findings indicate that BITC potentiates cell death and reduces the viability of CAR cells.

### 3.2. BITC triggers apoptosis and DNA breaks in CAR cells

**Fig. 3:**
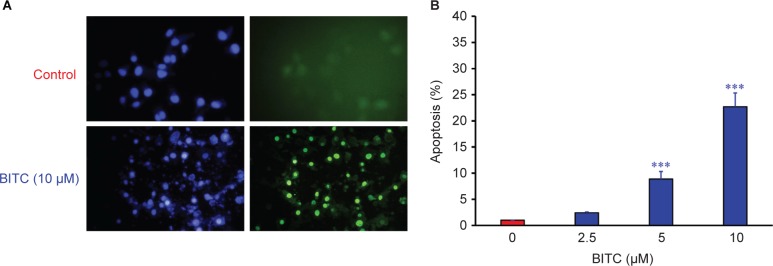
Effects of BITC on DAPI staining and TUNEL assay on CAR cells. Cells were harvested following treatment with or without 10 μM of BITC for 48 h, and 4’-6-diamidino-2-phenylindole (DAPI) solution (1 *μg/ml)* and an *In Situ* Cell Death Detection Kit, Fluorescein were used. (A) The image of TUNEL positive cells (green fluorescence) were shown in CAR cells after 10 μM BITC treatment for 48 h. (B) Apoptotic cells were qualified as described in the Materials and Methods section. The data are shown as the means ± SD (n = 3). ***p<0.05 compared to untreated cells.

To determine whether BITC-induced suppression of CAR cell viability was associated with apoptosis, we further examined the effects of BITC on nuclear morphology and DNA damage as evidenced through the use of DAPI staining and a terminal deoxynucleotidyl transferase dUTP nick end labeling (TUNEL) assay. The results showed that BITC increased the number of apoptotic CAR cells with DNA condensation (a characteristic of apoptosis) and generated more blue fluorescence, which means an increase in apoptotic cells. BITC also caused fragmented nuclei to form green fluorescence, indicating DNA breaks and cell apoptosis, when compared with untreated control cells (Fig. 3A). The quantitative data of BITC-induced apoptosis in CAR cells was carried out (Fig. 3B). The data showed that BITC strongly suppressed cell viability and induced apoptosis in CAR cells.

### 3.3. BITC elicits caspase-dependent apoptosis in CAR cells

**Fig. 4:**
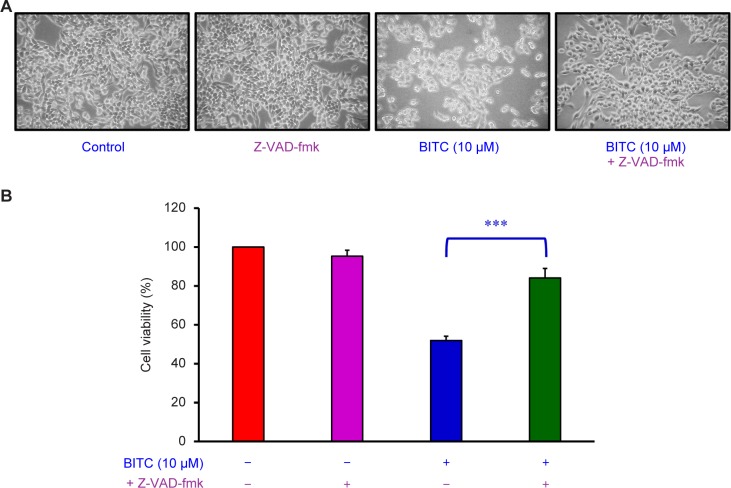
Effects of the pan-caspase inhibitor Z-VAD-fmk on apoptotic death in BITC-treated CAR cells. Cells were pretreated in the presence or absence of 10 μM Z-VAD-fmk and then exposed to 10 μM BITC for 48 h. (A) Cell morphologic observation was photographed. (B) Cell viability was assessed by MTT assay. The data are shown as the means ± SD (n = 3). ***p<0.05 compared to BITC-treated cells.

To further test whether the BITC-caused apoptosis is mediated through caspase cascade signaling, the CAR cells were preincubated with 10 μM Z-VAD-fmk (a pan-caspase inhibitor) and then treated with 10 μM BITC for 48 h. The results, gathered by a phase-contrast microscope, showed that Z-VAD-fmk significantly blocked BITC-induced apoptotic cell death and morphologic changes (Fig. 4A). In addition, Z-VAD-fmk reversed BITC-caused inhibition of cell viability in CAR cells (Fig. 4B). We can thus suggest that the apoptotic mechanism of BITC was involved in the caspase cascade pathway in CAR cells.

### 3.4. BITC-induced apoptosis is caspase-3/-9-dependent in CAR cells

**Fig. 5:**
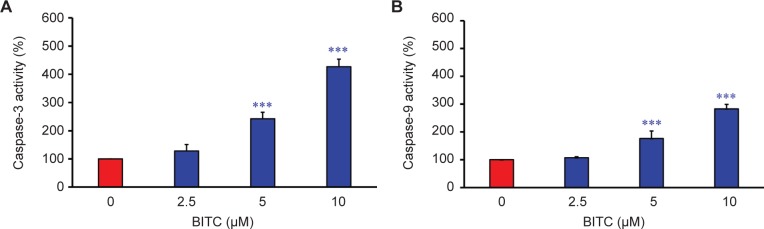
Effects of BITC on caspase-9 and caspase-3 activities in CAR cells. (A) caspase-3 and (B) caspase-9 activities were analyzed in CAR cells treated with 0, 2.5, 5, and 10 μM of BITC for 48 h. Data are presented as the means ± SD (n = 3). ***p < 0.001 versus untreated control.

We further investigated if BITC-induced apoptosis is medicated through an intrinsic pathway in CAR cells. Cells were treated with 2.5, 5, and 10 μM of BITC for 48 h, and the activities of caspase-3 and -9 were individually determined by a colorimetric assay. What we found was that BITC at 5 and 10 μM significantly stimulated caspase-3 activity in a concentration-dependent manner (Fig. 5A). Furthermore, the similar results also demonstrated that the promotion of caspase-9 activity was observed in BITC-treated CAR cells (Fig. 5B). Our results suggested that BITC induced apoptosis, and that the activation of caspase-9/-3 was involved in mitochondria-mediated apoptotic pathway in CAR cells.

### 3.5. BITC induced ROS production and loss of mitochondria membrane potential (Δψm) as well as altered the levels of apoptosis-related proteins in CAR cells.

**Fig. 6:**
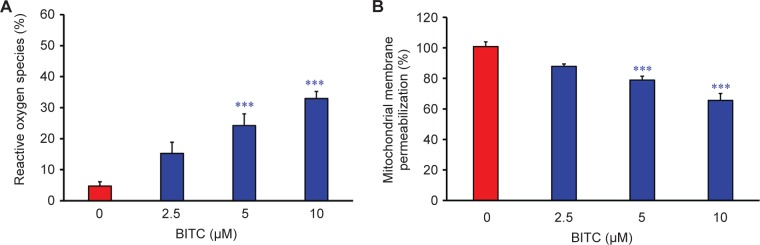
Effects of BITC on ROS production and mitochondrial membrane potential (Δψm) in CAR cells. Cells were incubated with 0, 2.5, 5, and 10 μM of BITC for 48 h. (A) ROS level was assessed by staining with H2DCFDA, and (B) loss of Δψm was measured with DiOC(3)6 by flow cytometry. Data are presented as the means ± SD (n = 3). ***p < 0.001 versus untreated control.

**Fig. 7:**
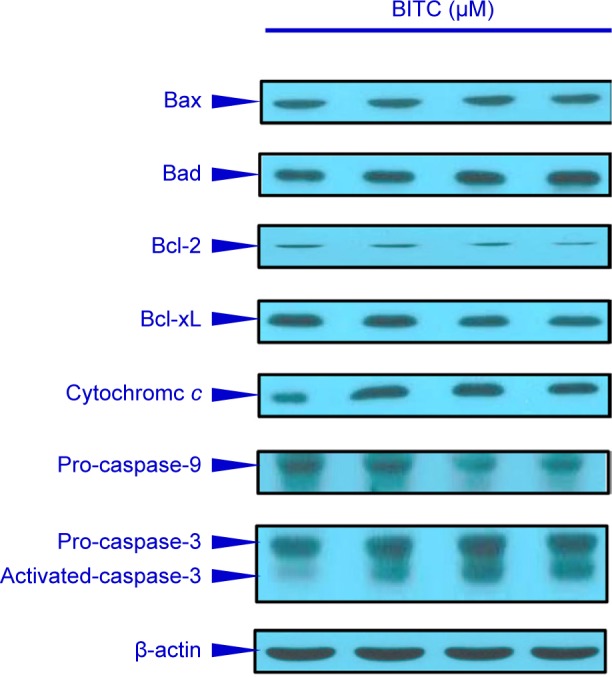
Effects of BITC on apoptotic signaling of CAR cells. Cells were treated without or with 2.5, 5, and 10 μM of BITC for 48 h, and cell lysates were collected and blotted using specific antibodies, including Bax, Bad, Bcl-2, Bcl-xL, cytochrome *c*, caspase-9 and caspase-3 by immunoblotting analysis as described in the Materials and Methods section. Each lane of protein signaling was normalized to β-actin.

**Fig. 8:**
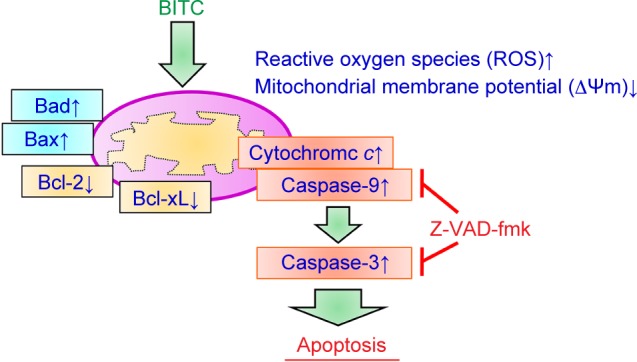
A proposed model of apoptosis-related signaling pathway induced by BITC in human cisplatin-resistant oral cancer CAR cells.

We have demonstrated that BITC induced apoptosis is caspase- 3/-9-dependent. To further investigate the upstream of associated signaling molecular in BITC-treated CAR cells, the cells were exposed to various concentrations (2.5, 5, and 10 μM ) of BITC for 24 h to detect the levels of ROS production and ΔΨm, which was done *via* a flow cytometric assay. Our results indicated that BITC promoted the production of ROS (Fig. 6A) as well as a loss of ΔΨm (Fig. 6B) in CAR cells in a concentration-dependent effect. Therefore, we suggest that mitochondrial dysfunction and ROS production contributed to BITC-induced caspase-3/-9-dependent apoptosis in CAR cells. To understand the mechanism of apoptosis in BITC-treated CAR cells, Bcl-2 family molecules and intrinsic signaling were determined by western blot. BITC at 2.5, 5, and 10 μM for 48 h increased the protein levels of Bax, Bad, and cytochrome c, but it decreased Bcl-2 and Bcl-xL in CAR cells (Fig. 7). Our findings from immunoblotting analysis indicated that BITC induced mitochondria-dependent apoptosis in CAR cells. Collectively, our results revealed that BITC modulated Bcl-2 family signaling and promoted the release of cytochrome *c* by activating intrinsic apoptotic cascade in CAR cells (Fig. 8).

## 4. Discussion

Many natural dietary foods and Chinese herbal medicines have been shown to exert obviously useful and therapeutic properties for human health [44]. The anticancer activity of isothiocyanates (ITCs) has been widely proofed [3, 44, 45]. In preclinical studies, the cancer-preventive efficacy of ITC treatments has been observed [6, 46, 47]. The anticancer mechanisms of ITC have been broadly investigated, including cell cycle arrest, anti-oxidation action, enhancement of DNA damage and repair, induction of apoptosis and autophagy, anti-metastasis, and elimination of cancer stem cells [6, 45, 46, 48]. Benzyl isothiocyanate (BITC) is one of the ITCs. BITC is a naturally-occurring constituent of cruciferous vegetable and found in *Alliaria petiolata,* pilu oil, cauliflower, cabbage, broccoli, and papaya seeds [1-3, 49, 50]. BITC has been found to induce apoptosis in various cancer cell lines, including breast cancer cells (MCF-7, HBL-100 and MDA-MB- 231) [51, 52], breast cancer stem cells (bCSC) [53], colon cancer cells (HCT-116) [54], gefitinib-resistant lung cancer cells (PC9/ BB4) [55], glioma cells (U87MG) [56], hepatocellular carcinoma cells (Bel 7402 and HLE) [57], osteosarcoma cells (U-2 OS) [11], lung cancer cells (A549, SK-MES-1 and H661) [58], melanoma cells (A375.S2) [59], epidermoid carcinoma cells (A431) [60], pancreatic cancer cells (L3.6pL, MIA-PaCa2, and Panc1) [61], prostate cancer cells (Rv1 and PC3) [9], gastric cancer cells (AGS) [12], and oral cancer cells (OC2) [62]. Yeh *et al.* [62] reported that BITC inhibited cell growth, promoted G2/M phase arrest, and triggered apoptosis of oral cancer OC2 cells, with a minimal toxicity to normal PBMCs. In addition, Sehrawat *et al.* [51] showed that BITC induced apoptosis in breast cancer cells but did not affect normal breast MCF-10A cells. Those studies suggest that BITC exerted non-toxicity in normal cells. The molecular mechanisms of BITC-induced cell death in cisplatin-resistant oral cancer CAR cells are not yet fully understood. Nevertheless, our results are in accordance with those of a study by Yeh *et al,* which demonstrated that BITC inhibited cell growth and triggered apoptosis of human oral cancer OC2 cells [62].

In said Yeh *et al’s* study, BITC-induced apoptosis was meditated by the reduction of Mcl-1 and Bcl-2 protein levels, disruption of mitochondria membrane potential (ΔΨm), and an increase of ROS production and PARP cleavage level in oral cancer OC2 cells [62]. Several studies have demonstrated that intracellular ROS and disruption of mitochondria membrane potential can effectively induce cancer cell death, including apoptosis and autophagy [63-65]. The disruption of mitochondrial function (loss of ΔΨm) and an increase of ROS production eventually leads to the apoptosis of cancer cells [9, 28, 31, 32]. Previous studies using brain glioblastoma cells (GBM-8401), prostate cancer cells (PC-3), breast cancer cells (MCF-7 and MDA-MB-361), melanoma cells (A375.S2), and osteogenic sarcoma cells (U-2 OS) have demonstrated that BITC-induced apoptosis is associated with the generation of ROS [9, 11, 61, 66]. Our results showed that 5-10 μM of BITC significantly inhibited the cell growth of cisplatin-resistant CAR cells (Fig. 1, Fig. 2, and Supplementary data). Significant DNA condensation, DNA fragmentation (Fig. 3), and caspase-3/-9 activation were observed in BITC-treated cells (Fig. 5A and B), indicating that BITC can induce caspase-dependent apoptosis in CAR cells. We investigated the possible role of ROS generation and mitochondrial function in BITC-induced apoptosis of CAR cells *via* flow cytometry following staining with H_2_DCF-DA and DiOC_6_(3) (specific detection of ROS and mitochondria membrane potential, respectively). The results in Fig. 6 show that BITC significantly induced ROS generation (Fig. 6A) and disruption of mitochondria membrane potential (Fig. 6B) in a concentration-dependent manner in CAR cells. Our findings showed that BITC significantly disrupted mitochondria membrane potential (Fig. 6B), aided the release of cytochrome c and caspase-9 (Fig. 7 and Fig. 5B) and then activated the caspase-3 (Fig. 7 and Fig. 5C) for apoptosis in CAR cells. Our results in this study suggest that BITC provokes caspase-dependent and mitochondriamediated apoptosis in CAR cells. What we found provides new insights into the oral anticancer activity of BITC in cisplatinresistant CAR cells.

## 5. Conclusions

Our results support the findings that BITC-caused intrinsic apoptosis is mediated through ROS production and mitochondrial dysfunction in CAR cells. The proposed integrated model of the molecular signaling induced by BITC in CAR cells is summarized in Fig. 8. As far as we are aware, this study is the first to show that BITC represents a promising candidate as an adjuvant treatment for oral anticancer, and it might be a potential agent for patients with drug-resistant oral cancer in the future.

## Conflicts of interest

The authors declare that they have no conflicts of interest.

## Supplementary video

Effects of BITC on cell confluence and cell growth in CAR cells. CAR cells were incubated with or without μ10 of BITC. The dynamic cell imaging was done at a 2-h interval up to 48 h by an IncuCyte ZOOM System instrument.
